# The Comparative Effects of Anakinra and Tocilizumab on Inflammation and Cerebral Vasospasm in an Experimental Subarachnoid Hemorrhage Model

**DOI:** 10.3390/medicina60122025

**Published:** 2024-12-09

**Authors:** Güven Kılıç, Berk Enes Engin, Amir Halabi, Cengiz Tuncer, Mehmet Ali Sungur, Merve Alpay, Adem Kurtuluş, Hakan Soylu, Ali Gök

**Affiliations:** 1 Department of Neurosurgery, Faculty of Medicine, Duzce University, Duzce 81100, Türkiye; drberkengin@gmail.com (B.E.E.); amrhalabi90@gmail.com (A.H.); cengiztuncer@gmail.com (C.T.); dr.ademkurtulus@gmail.com (A.K.); 2 Department of Biostatistics, Faculty of Medicine, Duzce University, Duzce 81100, Türkiye; malisungur@yahoo.com; 3 Department of Biochemistry and Molecular Biology, Faculty of Medicine, Duzce University, Duzce 81100, Türkiye; mervealpay@duzce.edu.tr; 4 Department of Histology and Embryology, Faculty of Medicine, Duzce University, Duzce 81100, Türkiye; hknsyl85@gmail.com; 5 Experimental Animals Application and Research Center, Duzce University, Duzce 81100, Türkiye; aligok@duzce.edu.tr

**Keywords:** subarachnoid hemorrhage, anakinra, tocilizumab, vasospasm, inflammation, interleukin-1, interleukin-6

## Abstract

*Objective*: Subarachnoid hemorrhage (SAH) is a life-threatening cerebrovascular condition that triggers a robust inflammatory response and cerebral vasospasm. This study aimed to evaluate the effects of anakinra, an interleukin-1 receptor antagonist, and tocilizumab, an interleukin-6 receptor antagonist, on inflammation and vasospasm in an experimental rat SAH model. *Methods*: Forty male Sprague Dawley rats (200–250 g) were randomly assigned to five groups: control, SAH, SAH + anakinra (ANA), SAH + tocilizumab (TCZ), and SAH + anakinra + tocilizumab (ANA+TCZ). SAH was induced by injecting non-heparinized arterial blood into the cisterna magna. Treatment groups received anakinra (50 mg/kg twice daily), tocilizumab (8 mg/kg once daily), or their combination for three days. Blood and cerebrospinal fluid (CSF) samples were analyzed for inflammatory markers (IL-1, IL-6, TNF-α, CRP), and histopathological evaluations were conducted to assess vasospasm and apoptosis. *Results*: SAH significantly increased pro-inflammatory cytokines (IL-1, IL-6, TNF-α, CRP) and fibrinogen levels in serum and CSF while reducing the basilar artery lumen diameter (*p* < 0.001). Anakinra and tocilizumab treatments significantly reduced inflammatory markers and vasospasm severity compared to the SAH group (*p* < 0.05). Combination therapy was more effective in reducing inflammation and vasospasm than either treatment alone (*p* < 0.05). Anakinra showed a stronger effect on IL-1 reduction, while tocilizumab was more effective in lowering IL-6 levels. The ANA+TCZ group exhibited a significant decrease in caspase activity, indicating reduced apoptosis (*p* < 0.05). *Conclusions*: Anakinra and tocilizumab effectively mitigated inflammation and vasospasm in an experimental SAH model, with combination therapy showing superior efficacy. These findings suggest that targeting both IL-1 and IL-6 pathways may be a promising therapeutic strategy for managing SAH complications. Further studies are warranted to evaluate long-term outcomes and clinical implications.

## 1. Introduction

Subarachnoid hemorrhage (SAH) is a neurologically serious and life-threatening clinical condition characterized by the leakage of blood into the subarachnoid space between the vessels on the surface of the brain due to rupture of intracranial aneurysms or trauma [1,2]. Globally, SAH accounts for approximately 5–10% of all strokes, with case fatality rates reaching up to 40% despite advances in surgical and medical interventions. Survivors often face significant long-term disabilities, making it a major public health concern [3]. SAH is characterized by sudden headaches, loss of consciousness, and neurological deficits [4]. Mortality rates due to SAH are still high despite early diagnosis and surgical interventions, and a large proportion of these patients face long-term neurologic sequelae. Cerebral vasospasm, one of the most common complications of SAH, begins several days after aneurysm rupture or trauma and, if left untreated, can lead to severe consequences such as delayed cerebral ischemia [5,6].

Vasospasm after SAH causes excessive contraction of smooth muscles in the arterial walls, resulting in decreased cerebral blood flow, which increases secondary brain damage and further increases mortality and morbidity [7]. Hemoglobin and erythrocyte breakdown products released after hemorrhage trigger a robust inflammatory response in the surrounding tissues. IL-1 and IL-6 are critical drivers of this inflammatory cascade. IL-1 initiates inflammation by activating endothelial cells and leukocytes, while IL-6 amplifies the response, promoting oxidative stress and endothelial dysfunction. These processes collectively contribute to cerebral vasospasm and secondary ischemic injury [8]. Important mediators of this inflammatory response include proinflammatory cytokines such as interleukin-1 (IL-1), interleukin-6 (IL-6), and tumor necrosis factor alpha (TNF-α) [9,10]. IL-1 inhibition targets the upstream triggers of inflammation, while IL-6 blockade prevents sustained inflammatory and oxidative stress responses. These complementary mechanisms justify the investigation of combination therapy as a potential strategy for treating SAH complications. These cytokines increase in both plasma and cerebrospinal fluid (CSF), triggering the inflammatory cycle. Oxidative stress and endothelial damage as a consequence of neuronal inflammation play a central role in the pathophysiology of vasospasm. In this context, pharmacologic agents targeting the role of cytokines in inflammatory processes have great potential to reduce secondary damage after SAH [11,12].

Tocilizumab, an IL-6 receptor antagonist, has demonstrated efficacy in reducing vasospasm, neuronal cell death, and microclot formation in preclinical SAH models [13]. A study by Croci et al. [14] highlighted the drug’s vascular protective effects, showing significant reductions in ischemic damage caused by microclots. Furthermore, another study [15] identified correlations between cerebrospinal fluid lipid profile changes, cerebral vasospasm, and IL-6 synthesis, emphasizing the pivotal role of IL-6 in SAH pathology. Similarly, anakinra, an IL-1 receptor antagonist, has shown promise in suppressing inflammation and reducing neuronal damage in preclinical brain injury models. Given this central role of inflammation, inhibition of IL-1 and IL-6 pathways may be an important strategy to control inflammation and vasospasm after SAH.

Targeting both IL-1 and IL-6 pathways provides a dual approach to interrupting the inflammatory cycle at multiple levels. IL-1 antagonism suppresses the initiation of inflammation, while IL-6 blockade prevents its perpetuation and downstream effects, such as oxidative stress and vascular dysfunction. These complementary mechanisms justify the investigation of combination therapy in this study. Anakinra is a recombinant form of human interleukin-1 receptor antagonist (IL-1Ra) that suppresses the inflammatory response by inhibiting the inflammatory effects of IL-1 [16,17]. In clinical trials, anakinra has been shown to be effective in suppressing inflammation after brain injury. Tocilizumab is a monoclonal antibody that blocks IL-6 receptors and has a potential role in reducing vascular and neuronal damage by preventing the proinflammatory and prooxidative effects of IL-6 [18,19]. Preclinical studies have shown promising results for tocilizumab in reducing vasospasm, neuronal cell death, and microclot formation in SAH models [20,21]. These findings underscore the importance of targeting IL-6 in SAH treatment strategies. This deficiency provides an important opportunity to better understand the efficacy of these two anti-inflammatory agents in the treatment of severe clinical conditions such as aneurysmal SAH. The aim of this study was to comparatively investigate the effects of anakinra and tocilizumab, separately and in combination, on the inflammatory response and vasospasm after experimentally induced SAH. Our study aims to shed light on the potential anti-inflammatory and neuroprotective effects of these two agents for future clinical applications.

The aim of this study was to comparatively investigate the effects of anakinra and tocilizumab, separately and in combination, on the inflammatory response and vasospasm after experimentally induced SAH. Building on previous findings, this study seeks to evaluate the potential of these therapies to improve outcomes and inform future clinical applications. This study aims to fill the gap in the literature regarding pharmacologic agents that can be used in the treatment of inflammation and vasospasm after SAH and aims to provide a new perspective on treatment strategies in this field.

## 2. Materials and Methods

This study was conducted in the Laboratory of Experimental Animals Research and Application Center of Düzce University, Düzce Faculty of Medicine, in accordance with the necessary permissions obtained from Düzce University Düzce Faculty of Medicine Experimental Animals Local Ethics Committee (Project No. E2-23-4294). During the study, the principles of laboratory animal care were strictly followed, and all animals were obtained from the Düzce University Experimental Animal Research and Application Center. All rats used in the experiment were housed under standard laboratory conditions before the experiment. Regular health checks were performed, and animal welfare was meticulously observed throughout the experiment. Male Sprague Dawley rats were chosen to minimize hormonal variations that could affect inflammatory responses and outcomes.

### 2.1. Study Design

A total of 40 male Sprague Dawley rats weighing 200–250 g and 3 months old were used in the study. This specific weight and age range was selected to ensure physiological consistency and represent mature adult rats, which are commonly used in cerebrovascular studies. These rats were randomly divided into five different groups. The first group was the control group, in which subarachnoid hemorrhage (SAH) was not induced and no treatment was administered. These rats were examined under normal conditions and considered healthy individuals. The second group consisted of rats in which only subarachnoid hemorrhage was induced and no treatment was administered to the rats in this group. This group was used as an untreated control group after SAH to create a disease model.

The third group consisted of rats that received tocilizumab treatment after subarachnoid hemorrhage was induced. Rats in this group received tocilizumab (8 mg/kg sc) after subarachnoid hemorrhage. The fourth group consisted of rats treated with anakinra after induction of subarachnoid hemorrhage, and rats in this group received anakinra (50 mg/kg sc).

Finally, the fifth group consisted of rats treated with both tocilizumab and anakinra. The rats in this group received tocilizumab and anakinra combination therapy after subarachnoid hemorrhage was induced. These groups were designed to evaluate the effects of different treatment modalities on inflammation and vasospasm after subarachnoid hemorrhage. A sham surgery control group was not included in this study due to the focus on evaluating treatment effects in confirmed SAH cases. Future studies will address this limitation by incorporating sham and vehicle-only controls.

### 2.2. Experimental Protocol

Experimental subarachnoid hemorrhage was performed in rats under general anesthesia. For anesthesia, 35 mg/kg ketamine and 5 mg/kg xylazine were administered subcutaneously. To induce subarachnoid hemorrhage, 0.1 mL of cerebrospinal fluid from the cisterna magna was replaced with the same amount of non-heparinized tail artery blood injected slowly over 2 min. This protocol was selected based on its reproducibility and established use in preclinical SAH models.

Immediately after subarachnoid hemorrhage, tocilizumab and anakinra were administered to the rats in the treatment groups. Tocilizumab was administered subcutaneously at a dose of 8 mg/kg, and a total of three doses were given every 24 h. Anakinra was subcutaneously administered at a dose of 50 mg/kg twice daily. The doses and administration regimens were chosen based on prior studies demonstrating their efficacy in reducing inflammation in rodent models of brain injury [22].

After the treatment period continued for three days, the rats were sacrificed. Blood and cerebrospinal fluid (CSF) samples obtained after sacrifice were subjected to various biochemical and histopathological examinations.

### 2.3. Biomarker Analysis

In the study, CRP, TNF-α, IL-1β, IL-6, and fibrinogen levels were examined in order to evaluate inflammatory cell degeneration/regeneration values in groups subjected to applications (anakinra, tocilizumab, combined, and saline) at different durations and doses in regular group rules (the SAH model was created) in rats. Parameters were measured in both CSF and blood serum. Blood samples (minimum volume 0.5 mL) were taken by venipuncture and collected in a biochemical test tube containing gel. After blood was taken, the test tube was kept for 15 min and then spun at 1800× *g* for 10 min. After separation from serum, samples were stored at −20 °C. All samples were stored at different time points out of the circuit for variable intervals of freshness and collected from −20 to −80 deep freezers until analysis time thanks to single-time operation.

Total CRP, TNF-α, IL-1β, IL-6, and fibrinogen levels in the studied serum and CSF samples were measured spectrophotometrically at 460 nm (OD) using the Rat-specific Enzymatic Analysis Kit (Cat. No. E0053Ra BTLab; E0764Ra BTLab; E0107Ra BTLab; E0135Ra BTLab; EA0056Ra BTLab, BTLab, Shanghai, China) purchased commercially in the market and the level established by following the kit protocol in the BIOTEK ELX800 microplate reader device (Thermo Fisher Scientific, MA, USA). These biomarkers were chosen to reflect the inflammatory and coagulative responses associated with SAH pathophysiology. The results were determined using a calibration curve set up with two-point calibration specifically for its analysis (using the Curvexpert program, version 2.7.3, Hyams Development) and the standard curvature of the reagent barcode.

### 2.4. Histopathologic Evaluation

For histopathological examination, sections 4 µm thick, taken from paraffin-embedded blocks and placed on standard glass slides, were incubated at 75 °C for 40 min in an oven to remove the paraffin from the tissue. Basilar artery sections were specifically chosen due to their susceptibility to vasospasm in SAH. After being removed from the oven, the sections were placed in xylene 1 and xylene 2 for 10 min each. The tissues were then dipped 10 times in 100%, 90%, 80%, and 70% ethanol, followed by a wash in running water to rehydrate the tissue.

### 2.5. Immunohistochemistry

The 4 µm sections were taken on polylysine-covered slides and deparaffinized and then rehydrated. Citrate buffer (pH 6.0) was used for antigen retrieval. The sections were boiled in a 750-watt microwave oven for 7 min. Immunohistochemical analysis focused on cleaved caspase-3 as a marker of apoptosis, given its established role in neuronal cell death.

### 2.6. Statistical Analysis

Statistical analyses were utilized with IBM SPSS v.22 (IBM Corp. Released 2013. IBM SPSS Statistics for Windows, Version 22.0. Armonk, NY, USA: IBM Corp.) package. The distribution of data was examined with the Shapiro–Wilk test, and the variance homogeneity was analyzed with the Levene test. One-way ANOVA and Welch tests were used in group comparisons. Tukey HSD and Dunnett’s *t* post hoc tests were used for multiple comparisons. Data were summarized with mean and standard deviation, and the statistical significance level was considered 0.05.

## 3. Results

After the three-day period, the rats were sacrificed, and blood and CSF samples were obtained to perform biochemical and histopathological examinations. The experimental design allowed for a detailed analysis of inflammatory and structural changes associated with SAH and the effects of different treatment regimens. There were significant differences in the SAH group compared to the control group for all parameters evaluated. All parameters were found to be significantly higher in the SAH group, except lumen diameter, which was significantly lower compared to the control (*p* < 0.001, Table 1).

**Figure 1:**
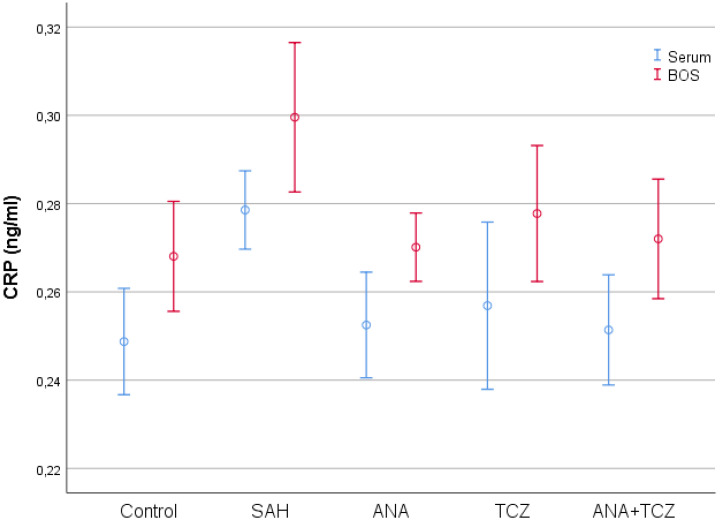
Comparison of serum and CSF CRP levels between groups. SAH: subarachnoid hemorrhage, ANA: anakinra, TCZ: tocilizumab, CSF: cerebrospinal fluid, CRP: C-reactive protein.

CRP levels both in serum (*p* = 0.019) and CSF (*p* = 0.008), IL-1 levels in serum (*p* = 0.004) and CSF (*p* < 0.001), and IL-6 levels in serum (*p* < 0.001) and CSF (*p* = 0.012) were significantly reduced in the ANA-administered group, TNF-α (*p* = 0.003) and FIB (*p* = 0.011) levels showed significant treatment effects only in CSF samples compared to the SAH group. No significant differences were found in serum levels of TNF-α (*p* = 0.171) and FIB (*p* = 0.372). Additionally, the lumen diameter was significantly higher in the ANA group than in the SAH group (*p* < 0.001), but caspase levels showed no significant difference, though a notable reduction was observed (*p* = 0.805). In comparison to the SAH group, the TCZ-administered group showed significant treatment effects in serum levels of IL-6 (*p* < 0.001) and CSF levels of TNF-α (*p* = 0.001), IL-1 (*p* = 0.034), IL-6 (*p* < 0.001), and FIB (*p* < 0.001). Despite a decrease after SAH, no significant differences were found in serum levels of CRP (*p* = 0.070), TNF-α (*p* = 0.183), IL-1 (*p* = 0.228), or FIB (*p* = 0.588), nor in CSF levels of CRP (*p* = 0.077). The TCZ group also demonstrated a significant increase in lumen diameter compared to the SAH group (*p* < 0.001), though no significant reduction in caspase levels was observed (*p* = 0.392). When the ANA+TCZ combination treatment group was compared to the SAH group, significant treatment effects were observed for nearly all parameters except for serum levels of TNF-α (*p* = 0.064) and FIB (*p* = 0.326). This group exhibited a superior reduction in inflammatory markers, including CSF levels of IL-1 and IL-6, as well as improved lumen diameter and reduced caspase levels. When comparing the efficacy of ANA and TCZ, no significant difference was observed in most parameters except for the CSF level of IL-1 (*p* = 0.002). However, although no statistically significant difference was detected between ANA and TCZ groups, ANA reduced IL-1 better in both serum and CSF, while TCZ reduced IL-6 better in both serum and CSF. TCZ also demonstrated a slightly greater effect on CSF fibrinogen compared to ANA, though this difference was not statistically significant. The ANA+TCZ combination group had a greater effect on several parameters, including CSF levels of IL-1 and IL-6, lumen diameter, and caspase level, compared to the individual ANA and TCZ groups. Significant differences were observed between the TCZ and ANA+TCZ groups in CSF IL-1 levels (*p* = 0.006) and between the ANA and ANA+TCZ groups in CSF IL-6 levels (*p* = 0.005). CRP values, which were statistically significantly increased in both serum and CSF with SAH compared to control, decreased in all treatment groups. These decreases were significant for the ANA and ANA+TCZ groups in both serum and CSF, while no significant decrease was seen for the TCZ group in either serum or CSF. No statistically significant difference was detected between treatment groups (Figure 1).

**Figure 2:**
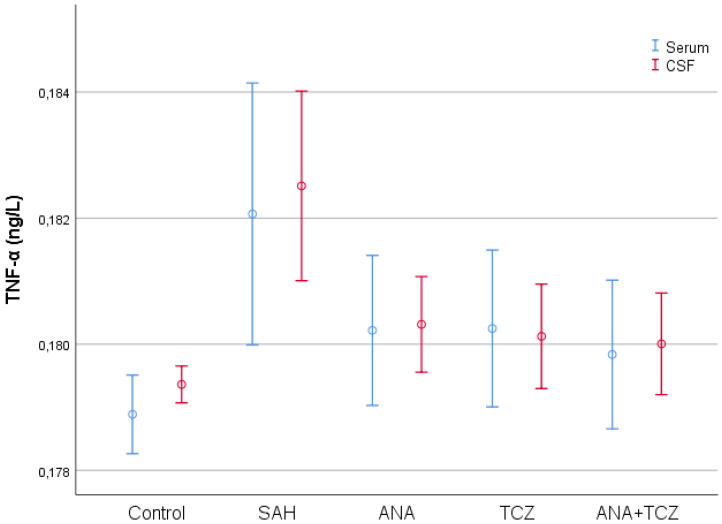
Comparison of serum and CSF TNF-α levels between groups.

TNF-α values, which were statistically significantly increased in both serum and CSF samples with SAH compared to control, decreased significantly only in CSF samples for all treatment groups. Although all treatment groups decreased TNF-α levels in serum samples, these reductions were not statistically significant. The decreases were significant for all treatment groups in CSF levels of TNF-α, and no differences were observed between ANA, TCZ, or ANA+TCZ treatments (Figure 2).

**Figure 3:**
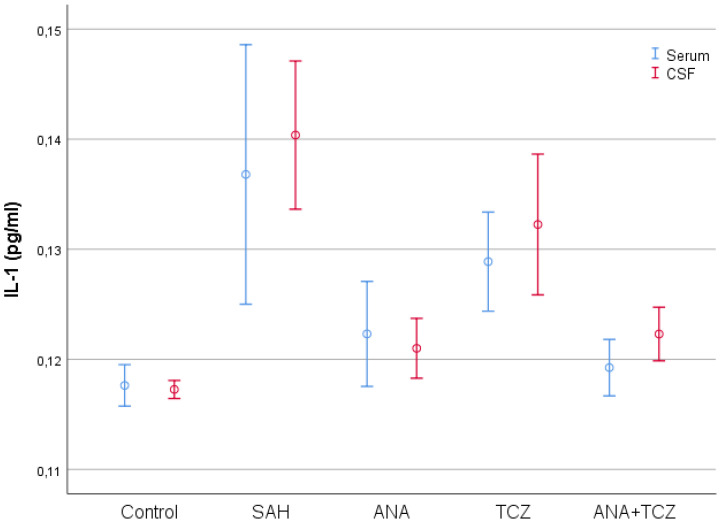
Comparison of serum and CSF IL-1 levels between groups. SAH: subarachnoid hemorrhage, ANA: anakinra, TCZ: tocilizumab, CSF: cerebrospinal fluid, IL-1: interleukin 1.

IL-1 values, which were statistically significantly increased in both serum and CSF samples with SAH compared to control, decreased significantly for all treatment groups in both serum and CSF samples, except for the TCZ group in serum samples. The ANA and ANA+TCZ groups showed significantly greater reductions in CSF IL-1 levels compared to TCZ, highlighting ANA’s more pronounced effect on this cytokine (Figure 3).

**Figure 4:**
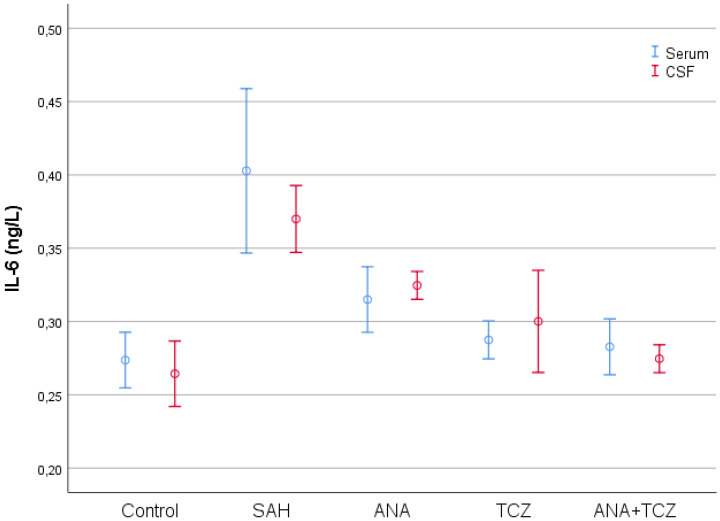
Comparison of serum and CSF IL-6 levels between groups. SAH: subarachnoid hemorrhage, ANA: anakinra, TCZ: tocilizumab, CSF: cerebrospinal fluid, IL-6: interleukin 6.

IL-6 values, which were statistically significantly increased in both serum and CSF samples with SAH compared to control, decreased significantly for all treatment groups in both serum and CSF samples. However, while the ANA group significantly reduced IL-6 levels compared to SAH, the decreases in the TCZ and ANA+TCZ groups were more substantial. The ANA+TCZ group showed the most pronounced reduction in CSF IL-6 levels, with significant differences observed between ANA and ANA+TCZ (*p* = 0.005) (Figure 4).

**Figure 5:**
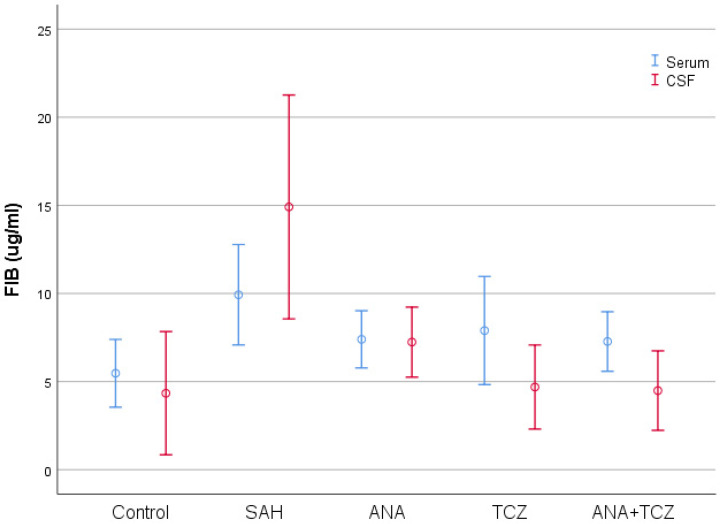
Comparison of serum and CSF FIB levels between groups. SAH: subarachnoid hemorrhage, ANA: anakinra, TCZ: tocilizumab, CSF: cerebrospinal fluid, FIB: fibrinogen.

Fibrinogen (FIB) values, which were statistically significantly increased in both serum and CSF with SAH compared to control, decreased significantly only in CSF samples for all treatment groups. Although all treatment groups decreased FIB levels in serum samples, these reductions were not statistically significant. The decreases in CSF FIB levels were similar across the ANA, TCZ, and ANA+TCZ groups (Figure 5).

**Figure 6:**
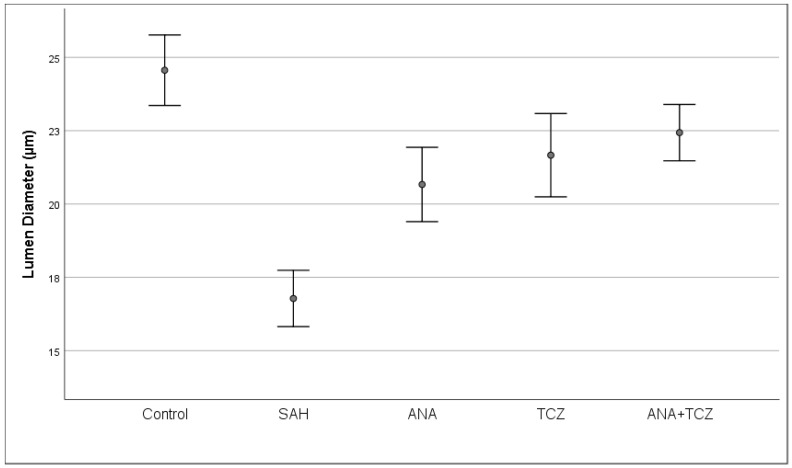
Comparison of lumen diameters between groups. SAH: subarachnoid hemorrhage, ANA: anakinra, TCZ: tocilizumab.

Lumen diameter, which was statistically significantly decreased with SAH compared to control, increased significantly in all treatment groups. No significant differences were observed between the ANA, TCZ, or ANA+TCZ groups for this parameter (Figure 6).

**Figure 7:**
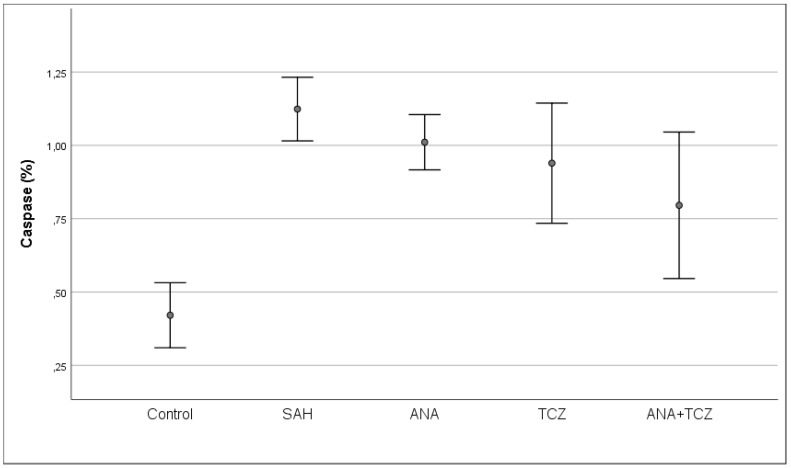
Comparison of caspase levels between groups. SAH: subarachnoid hemorrhage, ANA: anakinra, TCZ: tocilizumab.

Caspase levels, which were statistically significantly increased with SAH compared to control, decreased slightly in all treatment groups, but this decrease was only significant for the ANA+TCZ group. Despite this improvement, caspase levels in all treatment groups remained significantly higher than those in the control group (Figure 7).

## 4. Discussion

This study investigated the effects of treating the inflammatory response and cerebral vasospasm after subarachnoid hemorrhage with the interleukin-1 receptor antagonist anakinra and the interleukin-6 receptor antagonist tocilizumab. The results show that both agents have a significant effect on inflammatory biomarkers and that combination therapy, in particular, suppresses inflammation more potently. These findings align with previous studies highlighting the role of IL-1 and IL-6 in mediating inflammation and their impact on cerebral vasospasm after SAH [14,15]. These findings support cytokine inhibition as a potential therapeutic option in the management of inflammation after SAH.

Inflammation and vasospasm after subarachnoid hemorrhage are important factors determining the prognosis of patients [23,24]. SAH triggers a widespread inflammatory response in brain tissue, leading to neuronal damage. Proinflammatory cytokines, especially IL-1 and IL-6, are known to play a central role in this process [25,26]. In our study, significant increases in the levels of these cytokines in serum and cerebrospinal fluid were observed in the SAH group. These findings confirm that elevation of cytokines is one of the main causes of inflammatory response and vasospasm after SAH. This inflammatory cascade leads to endothelial dysfunction, oxidative stress, and microvascular thrombosis, further exacerbating neuronal injury [27,28].

Anakinra was found to be effective in suppressing inflammation after SAH by inhibiting the inflammatory effects of IL-1 [29]. Our study shows that anakinra significantly decreased IL-1 levels in both serum and CSF. The effect of IL-1 at the onset of the inflammatory process has become an important target for the prevention of neuronal damage after SAH. This is consistent with its established role in other models of neuroinflammation, such as traumatic brain injury and ischemic stroke [30,31]. Likewise, tocilizumab treatment, which targets the central role of IL-6 in inflammation, has also been shown to be effective. The significant decrease in IL-6 levels clearly demonstrates the suppressive effect of tocilizumab on inflammation. These results further corroborate findings from preclinical studies emphasizing the vascular protective effects of IL-6 inhibition.

Another remarkable finding in our study was that the combination of anakinra and tocilizumab was more effective than when both agents were used alone. This result suggests that combination therapy has a stronger effect in suppressing the inflammatory response after SAH. Combination therapy targeting both IL-1 and IL-6 pathways provides a more comprehensive suppression of inflammation by inhibiting various stages of inflammation [32,33]. Targeting upstream and downstream cytokine signaling pathways simultaneously likely accounts for the enhanced efficacy of combination therapy.

This finding suggests that combination therapy may be effective in improving prognosis after SAH.

Cerebral vasospasm is one of the most serious complications after SAH, leading to oxygenation disorders and increasing secondary brain damage. In our study, we observed a significant decrease in basilar artery lumen diameter narrowing in the treated groups [34,35]. It was observed that vasospasm was controlled more effectively, especially with combination therapy. These results suggest that cytokine inhibition not only suppresses inflammation but also reduces the severity of cerebral vasospasm. This dual benefit of reducing inflammation and vasospasm underscores the potential of cytokine inhibitors as neuroprotective agents. Moreover, IL-6 inhibition has been shown to reduce microclot formation, a critical factor in post-SAH ischemic damage [36]. This reduction in the severity of vasospasm is an important finding in terms of reducing neurological damage and improving prognosis.

This study has some limitations. First of all, the animal model used experimentally in this study may not completely reflect the pathophysiology after SAH in humans. Further studies are needed to determine the extent to which the findings obtained under experimental conditions can be transferred to clinical applications. The anatomical and physiological differences between rodents and humans, particularly in vascular reactivity and immune responses, must be considered when interpreting these results. Furthermore, our study focused on short-term results, and longer follow-up studies are required to evaluate long-term effects. Factors such as the long-term neuroprotective effects of both anakinra and tocilizumab treatments and potential side effects of combination therapy should be addressed in more detail in future research.

Another limitation is the limited number of biomarkers used to assess inflammation and vasospasm. A more comprehensive analysis of the different components of inflammation with more biochemical parameters may better reveal the efficacy of treatment strategies. Finally, only male rats were used in the study; the effect of sex differences on response to treatment could not be assessed. Sex-specific responses to inflammation and treatment have been reported in other models, warranting future studies in both male and female cohorts [37]. Both sex differences and different age groups should be considered in future studies.

## 5. Conclusions

This study shows that inflammation and cerebral vasospasm following SAH can be treated with the interleukin-1 receptor antagonist anakinra and the interleukin-6 receptor antagonist tocilizumab. Both therapeutic agents effectively suppressed the inflammatory response, and the combination therapy showed a stronger effect compared to monotherapy. It has been demonstrated that cytokine inhibition may be a potential therapeutic strategy for the management of inflammation after SAH. The observed synergistic effects of IL-1 and IL-6 inhibition provide a compelling rationale for further exploration of this combination in clinical settings. However, further clinical studies are needed to translate these findings into clinical practice. Future studies should more comprehensively examine the effects of long-term treatment approaches to cytokine inhibition and combination therapies on neurologic prognosis. Additionally, studies should investigate the impact of cytokine inhibition on other critical aspects of SAH pathology, such as blood-brain barrier integrity and neurovascular coupling.

## Figures and Tables

**Table 1:** Comparison of biochemical parameters measured in serum and CSF samples.

	Control (*n* = 8)	SAH (*n* = 8)	ANA (*n* = 8)	TCZ (*n* = 8)	ANA+TCZ (*n* = 8)	*p*
Serum CRP (ng/mL)	0.2487 ± 0.0144 ^a^	0.2786 ± 0.0106 ^b^	0.2525 ± 0.0143 ^a^	0.2569 ± 0.0226 ^ab^	0.2514 ± 0.0149 ^a^	**0.004**
Serum TNF-α (ng/L)	0.1789 ± 0.0007 ^a^	0.1821 ± 0.0025 ^b^	0.1802 ± 0.0014 ^ab^	0.1803 ± 0.0015 ^ab^	0.1798 ± 0.0014 ^ab^	**0.008**
Serum IL-1 (pg/mL)	0.1176 ± 0.0023 ^a^	0.1368 ± 0.0141 ^c^	0.1223 ± 0.0057 ^ab^	0.1289 ± 0.0054 ^bc^	0.1192 ± 0.0031 ^ab^	**<0.001**
Serum IL-6 (ng/L)	0.2737 ± 0.0227 ^a^	0.4028 ± 0.0671 ^b^	0.3150 ± 0.0268 ^a^	0.2875 ± 0.0155 ^a^	0.2828 ± 0.0228 ^a^	**<0.001**
Serum FIB (µg/mL)	5.4680 ± 2.3009 ^a^	9.9280 ± 3.4066 ^b^	7.3954 ± 1.9423 ^ab^	7.8955 ± 3.6719 ^ab^	7.2732 ± 2.0222 ^ab^	**0.048**
CSF CRP (ng/mL)	0.2681 ± 0.0149 ^a^	0.2996 ± 0.0203 ^b^	0.2701 ± 0.0093 ^a^	0.2777 ± 0.0184 ^ab^	0.2720 ± 0.0162 ^a^	**0.003**
CSF TNF-α (ng/L)	0.1794 ± 0.0003 ^a^	0.1825 ± 0.0018 ^b^	0.1803 ± 0.0009 ^a^	0.1801 ± 0.0010 ^a^	0.1800 ± 0.0010 ^a^	**<0.001**
CSF IL-1 (pg/mL)	0.1173 ± 0.0010 ^a^	0.1404 ± 0.0081 ^c^	0.1210 ± 0.0033 ^a^	0.1323 ± 0.0076 ^b^	0.1223 ± 0.0029 ^a^	**<0.001**
CSF IL-6 (ng/L)	0.2644 ± 0.0267 ^a^	0.3700 ± 0.0273 ^c^	0.3246 ± 0.0114 ^b^	0.3001 ± 0.0417 ^ab^	0.2746 ± 0.0114 ^a^	**<0.001**
CSF FIB (µg/mL)	4.3375 ± 4.1829 ^a^	14.910 ± 7.5979 ^b^	7.2388 ± 2.3763 ^a^	4.6888 ± 2.8536 ^a^	4.4856 ± 2.6973 ^a^	**<0.001**
Lumen diameter (µm)	24.5610 ± 1.4389 ^a^	16.7790 ± 1.1489 ^c^	20.6656 ± 1.5151 ^b^	21.6643 ± 1.6980 ^b^	22.4331 ± 1.1484 ^b^	**<0.001**
Caspase (%)	0.4209 ± 0.1326 ^a^	1.1238 ± 0.1174 ^c^	1.0108 ± 0.1126 ^bc^	0.9393 ± 0.2451 ^bc^	0.7958 ± 0.2986 ^b^	**<0.001**

SAH: subarachnoid hemorrhage, ANA: anakinra, TCZ: tocilizumab, CRP: C-reactive protein, TNF-α: tumor necrosis factor-alpha, IL: interleukin, FIB: fibrinogen, CSF: cerebrospinal fluid, different superscript letters denote significant differences between the groups according to the post hoc test.

## Data Availability

The data that support the findings of this study are available from the corresponding author upon reasonable request.
